# From Veterinary Medicine to Illicit Drug Supply: Utilising Social Media to Explore the Rising Emergence of Veterinary Medicines in Human Health

**DOI:** 10.3390/brainsci15020172

**Published:** 2025-02-10

**Authors:** Josie Dunn, Fabrizio Schifano, Ed Dudley, Davide Arillotta, Amira Guirguis

**Affiliations:** 1 Pharmacy, Medical School, The Grove, Swansea University, Swansea SA2 8PP, UK; 2009986@swansea.ac.uk; 2 Psychopharmacology, Drug Misuse and Novel Psychoactive Substances Research Unit, School of Life and Medical Sciences, University of Hertfordshire, Hatfield AL10 9AB, UK; f.schifano@herts.ac.uk (F.S.); davide.arillotta@yahoo.it (D.A.); 3 Medical School, The Grove, Swansea University, Swansea SA2 8PP, UK; e.dudley@swansea.ac.uk; 4 Department of Neurosciences, Psychology, Drug Research and Child Health, Section of Pharmacology and Toxicology, University of Florence, Viale G. Pieraccini, 6, 50139 Florence, Italy

**Keywords:** veterinary drugs, animal drugs, substance use, misuse, social media, netnography

## Abstract

Background/Objectives: The misuse of veterinary drugs is a growing concern, with increasing evidence of their presence in illicit drug markets and their use as alternatives to traditional substances. Methods: This study explores Reddit discussions on the misuse of veterinary drugs on Reddit, focusing on xylazine, carfentanil, medetomidine, pentobarbital, phenylbutazone, and acepromazine. Reddit was utilised for its abundant real-time data on users’ thoughts and experiences with substance misuse. Through a combination of manual and Artificial Intelligence (AI)-driven thematic analysis, we examined posts and comments to explore patterns of misuse. Results: The themes analysed included adverse effects, polysubstance misuse, routes of administration, motivations for misuse, and methods of obtaining these drugs. Our findings revealed that xylazine, medetomidine, carfentanil, and pentobarbital exhibit significant potential for misuse, while phenylbutazone and acepromazine are not widely misused. Despite this, phenylbutazone and acepromazine have been identified as adulterants in the illicit drug supply in the United States. The most discussed themes included motivations for misuse, followed by public experiences and perceptions, as well as adverse effects. Conclusions: The dual-method approach of combining manual interpretation with AI analysis allowed for a comprehensive understanding of social media discussions. This research highlights the importance of monitoring online platforms for early indicators of emerging drug trends, offering valuable insights to inform public health policies and intervention strategies. Impact Statement: This research highlights the growing public health risk posed by veterinary drug misuse, underscoring the need for enhanced monitoring, regulatory frameworks, and education to address their diversion into illicit markets. By leveraging social media as an early detection tool for emerging drug trends, our findings can inform targeted interventions.

## 1. Introduction

In 2022, England and Wales experienced the highest number of deaths related to drug poisoning since 1993, with an increased rate every year since 2012 [1]. This growing issue could be attributed to the ever-growing drug market, targeting problematic users who are constantly chasing their next “high”. As the drug market expands globally, scientific knowledge regarding the potency, purity, and combinations of drugs and their health impacts remains limited [2]. In 2021, drug misuse in the veterinary setting was described as an “under-recognised avenue”, where veterinarians were represented often overlooked as a source of prescription drug misuse [3]. This oversight persists despite their ability to prescribe, administer, stock, and dispense drugs with misuse potential [4]. Individuals with substance use disorders frequently attempt to acquire medications to satisfy their cravings, with reports of intentional pain inflicted on animals to obtain veterinary analgesics [5]. Accessing multiple prescriptions from various veterinary clinics (vet shopping) has been observed, where the number of patients receiving any class of controlled substances from four or more veterinarians increased three-fold between 2014 and 2019 [6].

Human misuse of veterinary medications is well documented, with reports of xylazine exposure in humans dating back to the 1970s [7]. However, in recent years, incidents involving this potent veterinary tranquiliser have surged, particularly in the United States (US), where the South saw a 193% rise in xylazine identifications from 2020 to 2021 [8]. This pattern has now emerged in the United Kingdom (UK), where xylazine was detected in sixteen individuals, including eleven fatalities [9]. Between May 2022 and August 2023, xylazine was frequently detected in the UK in combination with other drugs of misuse, including opioids (heroin, fentanyl), benzodiazepines (bromazolam), and other drugs of misuse, such as ketamine and pregabalin [9]. Although data on its misuse in the UK remain limited, the Welsh Emerging Drugs and Identification of Novel Substances (WEDINOS) identified xylazine in over 50 samples between January 2020 and June 2024, with all cases involving xylazine as an unintended component of the purchased substance [10]. Notably, xylazine was detected for the first time in two THC vape samples in 2022, demonstrating its growing presence and potential risks as an adulterant [10].

Medetomidine, a veterinary non-selective alpha-2-agonist like xylazine, has also been gaining recognition as an adulterant across the recreational opioid drug supply in the US. It is yet to be identified in the UK illicit supply (as of July 2024), yet it has been reported to be rapidly proliferating across the USA and Canada in samples also containing heroin, fentanyl, xylazine, and cocaine [11,12]. Medetomidine’s increasing prevalence is concerning due to it being a more potent, selective, and specific a-2-agonist, exhibiting increased sedation compared with xylazine [13]. Alongside medetomidine, two other veterinary medications—acepromazine (a phenothiazine) and phenylbutazone (a non-steroidal anti-inflammatory drug (NSAID))—were identified as potential toxic adulterants and predicted to be the “next xylazine” [14]. Although acepromazine reports remain relatively low, phenylbutazone has been identified in 116 seized drug samples between 2016 and 2021, alongside heroin, fentanyl, xylazine, tramadol, and cocaine [15]. Pentobarbital, a veterinary euthanasia agent, has also been detected in seized samples across the US [15,16], although it is classified as a Class B Schedule 3 drug. These five drugs’ pharmacological profiles have made them valuable tools in veterinary medicine, yet it is these same properties that influence the dangerous outcomes in humans. The lack of control or scheduling of these drugs may contribute to their increased availability and prevalence, leading to their rise as adulterants in the illicit opioid supply.

Carfentanil is described as an ultrapotent, selective agonist of the μ-opioid receptor, with a potency 100× greater than fentanyl [17]. In 2022, 14 countries reported seizing a total of 6.5 kg of carfentanil, accounting for 273 seizures across Europe [2]. Although this represents a slight decrease from the 308 seizures reported in 2020 [18], carfentanil misuse is believed to be underreported due to its exclusion from routine screenings and limited information on its abuse liability and dependence [19]. Unlike other veterinary medications, carfentanil is scheduled under the Misuse of Drugs Act 1971 and the Misuse of Drugs Regulations 2001 as a Class A, Schedule 2 substance [20]. Despite its classification, it is crucial to allocate attention and effort to prevent another spike in overdose cases due to its extreme potency.

Due to the relatively new emergence of these veterinary medications as adulterants, research needs to be conducted to gain novel and valuable information regarding their misuse and prevalence. This study aimed to employ a social media listening method to retrieve real-life data of user’s thoughts and experiences related to the selected drugs of interest.

## 2. Methodology

The social media platform Reddit has 52 million daily active users and over 138,000 active topical communities known as “subreddits” [21] and was chosen to be analysed for this study. Reddit offers a unique opportunity for anonymous discussions, which is particularly valuable when researching sensitive topics such as drug misuse. Users can freely share experiences and advice without the fear of stigma, a concern often present in face-to-face interviews or surveys. While traditional studies involving patients provide valuable insights, they are not always feasible or efficient for capturing the diverse perspectives and emergent trends seen in online communities. As such, Reddit serves as an accessible and rich data source for understanding contemporary issues in drug misuse. Although a netnographic study analysing veterinary drugs has never been conducted, prompts utilised for this analysis were adapted from previous research conducted on the topic of drug misuse [22].

### 2.1. Primary Data Search

**Figure 1:**
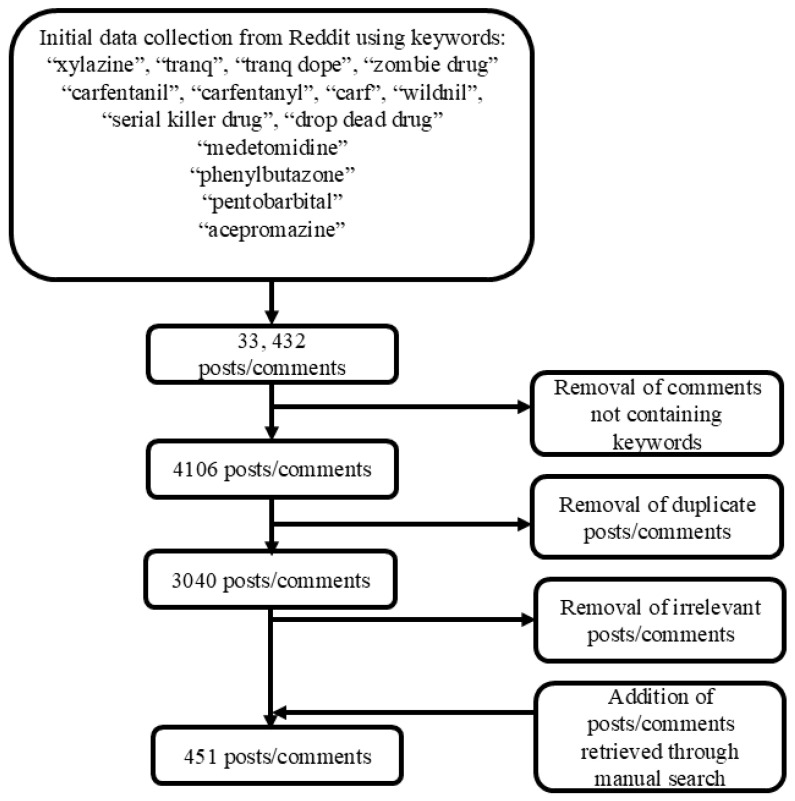
A schematic flowchart outlining the data collection process of extracting and screening Reddit posts and comments for analysis.

Initial research was conducted to identify the main slang words for the drugs of interest (e.g., “tranq dope”), given the use of social media as a source of data collection. No street names for medetomidine, phenylbutazone, pentobarbital, and acepromazine were identified. Reddit posts and comments were collected and screened using the web-scraping software Apify [23] between June and July 2024 (Figure 1). Data collection steps included the following:

- Specific search terms for each drug were used to retrieve the most relevant posts and comments (see Table 1);
- The scraper was set to search across posts, comments, communities, and users;
- The search included NSFW (Not Safe for Work) content, which covers explicit or inappropriate material, including drug misuse discussions;
- A maximum post and comment count of 9999 was set to ensure all available data were collected;
- No specific subreddits were selected in advance; Apify retrieved all threads and comments containing the chosen keywords without filtering by subreddit;
- The raw data were downloaded as an Excel file using Apify and imported into Microsoft Excel (Version 16.86 (24060916)).

Inclusion and exclusion criteria for Reddit posts and comments are detailed in Table 2.

### 2.2. Data Screening and Cleaning

The data cleaning and screening processes were performed without any AI contribution but with aid from computer software such as Excel and Apify. Any posts or comments not containing the specific keywords were removed. Duplicate posts and comments were also removed using the “remove duplicates” function on Excel. The remaining posts and comments were then screened for relevance to the research aims, and any piece of data that did not fit the inclusion criteria was removed. The data screening process was conducted by researcher JD, and regular discussions were held with senior author AG to ensure consistency and agreement on the inclusion and exclusion criteria. Although specific keywords were entered into Apify for data collection, a review of the extracted dataset in Excel revealed duplicate entries, demonstrating a possible error with the Apify software, where these duplicates likely resulted from repeated instances of the same content. While specific keywords were used to retrieve posts and comments, Apify also captured posts where the comments contained these keywords, even if the posts themselves did not. This led to the inclusion of unrelated content, such as posts about war, as the keyword “tranq” was often associated with guns in video games. As a result, additional filtering was required to exclude irrelevant posts and ensure that the dataset was accurate for the intended purpose of the study.

### 2.3. Data Analyses

For each of the selected keywords, the most prevalent themes identified were analysed manually. Themes were identified through an inductive approach, emerging naturally from the data rather than being predefined. A colour-coding method was utilised to distinguish between the various posts and comments within each theme. Any data that fell into multiple themes were included in each associated theme. A separate, manual search of Reddit posts and comments was also conducted to ensure Apify did not miss important data. This was performed by taking the keywords used in the scraping method and inputting them into the Reddit search bar. This manual search resulted in an additional 49 posts and comments that were not captured through the Apify tool. By analysing the most represented posts and comments on Reddit for relevance to the study, the posts and comments that met the inclusion criteria and were not captured by Apify were then incorporated into the study and put into their according themes. If a post or comment contained references to multiple drugs of interest, it was attributed to the drug that was specified as the keyword during the data retrieval in Apify.

### 2.4. Artificial Intelligence Analysis

In addition to conducting a manual thematic analysis, AI tools were employed to enhance the analysis process, using the two software programs numerous.ai [24] and ChatGPT [25]. The AI method was integrated to minimise researcher bias during the manual thematic analysis [26] and act as a complementary method to provide an additional validation layer. The process began by extracting posts and comments related to each drug, which were then sampled in groups of ten on Google Sheets [27]. These data were then inputted to numerous.ai with the following prompt: [=AI(“Please act as an expert in qualitative content analysis with a focus on public health issues. Analyse the following text specifically for themes related to the misuse of drugs. Identify these themes and present them in bullet points. Also, consider any biases or contextual factors that may impact the analysis. Ensure that the analysis is conducted with sensitivity and neutrality.”)].

Numerous.ai was used to conduct an initial thematic extraction of the content, ensuring that the AI’s identification of themes was based on patterns within the data that the manual analysis might have overlooked. These themes were then exported into Windows NotePad (Microsoft, 2024) documents in preparation for the ChatGPT analysis.

For further refinement, the thematic output from numerous.ai was grouped and analysed using ChatGPT in sets of 20. The following term was inputted into ChatGPT: [“I have a chunk of thematic analysis data for (drug name). Each post has several bullet points summarising themes and biases. Please analyse the following data and identify the top 5 most common themes and biases, ranked in order of prevalence.”].

ChatGPT was tasked with reviewing the themes identified using numerous.ai and cross-referencing them to provide a second layer of analysis, offering insights into the relative importance of each theme, as well as any contextual factors or biases present in the data. The responses from ChatGPT were captured into an Excel spreadsheet, where the themes identified by both AI tools were compared against the manual thematic analysis. The unique contributions of the AI tools allowed for a large amount of data to be processed quickly to identify patterns, validate findings, and add depth and consistency to the overall analysis process.

### 2.5. Ethical Considerations

Ethical approval was not required for this study as all the Reddit data was publicly available. However, ethical considerations regarding the use of public data were adhered to in this study. The identity of the users remained anonymous throughout the entirety of the study, with a focus on ensuring secure data handling and confidentiality. No attempts to identify or trace the users were made from the anonymous data, and only the data necessary for this research was extracted to respect user privacy. To minimise any potential impact on the users who wrote the extracted posts, no negative stereotypes or harmful generalisations will be concluded, thereby avoiding any stigma associated with drug misuse. Ethical approval for this study was granted by the Department of Pharmacy Ethics Committee at the University of Hertfordshire (protocol number aLMS/SF/UH/02951(5)).

## 3. Results

### 3.1. Manual Analysis

**Figure 2:**
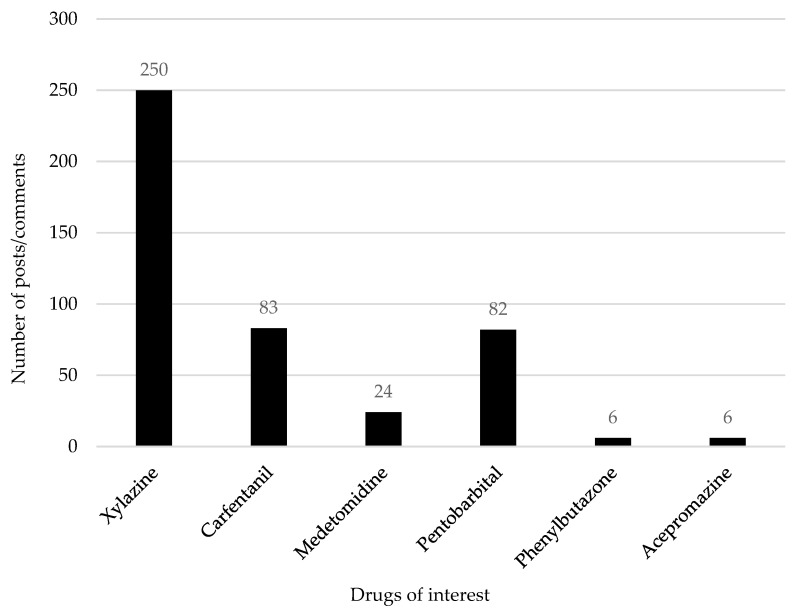
A bar graph showing the number of posts and comments retrieved through the manual search of keywords related to each substance, illustrating the volume of relevant content identified for each drug through the manual search.

**Figure 3:**
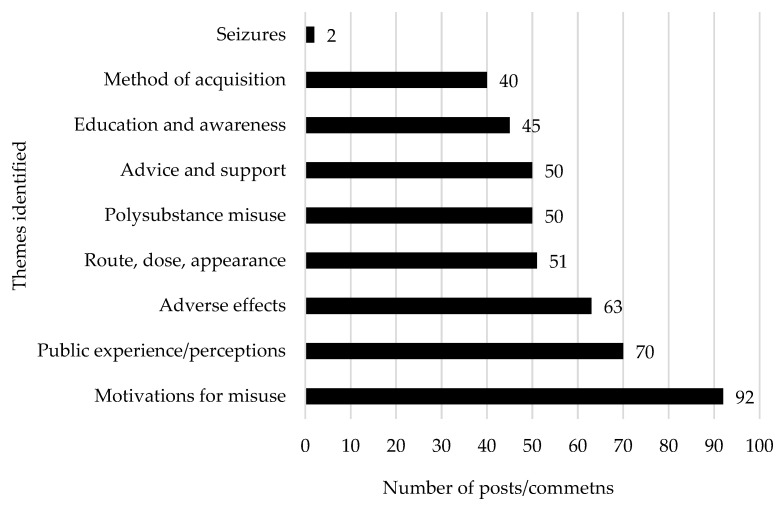
A clustered bar chart displaying the frequency of key themes identified in the study, along with the number of associated posts and comments.

After the collected data were screened, 451 posts/comments were deemed relevant for this study. Figure 2 illustrates the number of relevant posts and comments retrieved for each specific substance, with xylazine generating the highest number of entries, followed by carfentanil, pentobarbital, medetomidine, phenylbutazone, and acepromazine. Figure 3 presents the most prevalent themes encountered within the manual thematic analysis. The theme “motivations for misuse” was most frequently discussed, followed by “public experience/perceptions” and “adverse effects”. Other prominent themes included “route, dose, and appearance”, “polysubstance misuse”, and “advice and support”.

Both positive and negative posts and comments regarding veterinary drugs were encountered, with users often demonstrating why they like misusing these particular drugs and the motivations behind their misuse. On the other hand, other Reddit users came to social media to display their worries and concerns about the growing trend of veterinary misuse. For example, a handful of positive xylazine-related posts included *“Yes I enjoy tranq”* and *“I prefer xylazine”*, with users describing the reasons for the rise in misuse due to *“tranq gives fent legs…it helps fent subjectively last longer”,* with the longer high experienced making users *“feel more like you got your money’s worth”.* Carfentanil was described as *“the most euphoric substance”,* with a user displaying a desire for the substance, stating, *“I need some of that carfentanyl right now”.* Pentobarbital was described as *“pleasant and abusable”* and as a *“more intense high overall and lasted for a longer duration of time*”, as well as *“the holy grail”, “glorious”,* and *“blissful”.*

Conversely, comments such as *“evil”* and *“I hate tranq”* were used to describe xylazine. The term *“poison”* was also used to describe xylazine and medetomidine, although one user demonstrated a liking towards medetomidine, stating it was *“the best dope I’ve ever seen it had that met stuff in”.* Despite the positive comments about pentobarbital, there were many posts and comments where users described their intentions for use as suicidal intentions (e.g., *“I want to get access to pentobarbital and sodium thiopental, to take it and die”*), where one user described it as *“the gold standard for a quick and painless suicide”.* Whilst there were very few comments regarding the misuse of acepromazine and phenylbutazone, a user stated they misused phenylbutazone due to joint pain. The posts associated with the adverse effects of veterinary drugs were more commonly for xylazine and carfentanil, where xylazine was described to *“destroy any part of your body you put it into*” and carfentanil to make a user feel as if they *“should have been dead”* and having *“no idea how I’m still here”.* Polysubstance misuse data were retrieved from the study for five of the six veterinary drugs, with various dangerous combinations being identified. Xylazine was posted to be found in a sample with *“Lidocaine, Fentanyl, Tramadol, DXM, Niacinamide”* and *“Xanax and mdma”.* A harmful sample, named *“smurf dope”*, was noted to include “*either methamphetamine or heroin that has been laced with fentanyl. Perhaps not just fentanyl but carfentanil*”. In addition to this, a different sample, *“Super Mario”,* was described to contain *“xylazine, fentanyl, DPH, heroin, carfentanil”.* Different routes of administration were encountered in the study, including intravenous (carfentanil, xylazine), insufflation (xylazine), inhalation (xylazine), and oral ingestion (xylazine, pentobarbital).

Table 3 provides a summary of selected posts and comments with their corresponding drug and theme chosen. The brand name for pentobarbital, Nembutal, was often used by users on Reddit when discussing pentobarbital, despite it not being a keyword used in the data collection. It is important to note that Table 3 may include spelling errors and informal language, reflecting its social media origin.

### 3.2. AI-Driven Thematic Analysis

The two AI software programs, Numerous.ai and ChatGPT, were utilised to obtain the top occurring themes for each veterinary drug. Each drug was analysed independently, and the top five themes for each drug are summarised in Table 4. Although Numerous.ai applied filters to remove content related to suicide or promoting drug use, some relevant posts remained in the dataset, such as those associated with the suicidal intent of using pentobarbital.

## 4. Discussion

To our knowledge, this is the first study to use social media as a source of novel insights into the growing misuse of veterinary drugs in human health. By applying AI-driven thematic analysis, the study identified key themes in Reddit discussions related to veterinary drug misuse. The selected drugs—xylazine, carfentanil, medetomidine, pentobarbital, phenylbutazone, and acepromazine—were investigated based on the group’s prior research, which identified them as emerging public health threats [28,29].

Of the six drugs analysed in this study, only carfentanil and pentobarbital are classified as controlled substances under Schedules 1 and 3, respectively, in the Misuse of Drugs Regulations 2001 [20]. Xylazine, medetomidine, phenylbutazone, and acepromazine remain unregulated. Despite xylazine’s recognition as a public health threat in the UK due to its infiltration into the illicit drug market [30], no regulatory measures have been established. By August 2023, xylazine was detected in 35 cases in the UK through toxicology, drug screening, and seizure methods, with 11 fatalities reported [9]. In the US, xylazine detection continues to rise, becoming the most frequently identified adulterant with a positivity rate of 15.8%, nearly double the 8% rate in 2018 [31].

Although medetomidine misuse has not been documented in the UK, its emergence in the US has alarmed healthcare professionals. In May 2024, a public alert was issued due to increasing hospitalisations and overdoses [12]. Medetomidine was found alongside fentanyl, xylazine, and heroin in several states, but its misuse may be under-reported due to limited testing and detection capabilities.

Phenylbutazone, a non-steroidal anti-inflammatory drug (NSAID), has been detected as an adulterant in 116 drug samples seized in Pennsylvania, appearing alongside heroin, fentanyl, xylazine, and cocaine [32]. Acepromazine, widely used in veterinary pre-anaesthetic procedures, was highlighted by the Centre for Forensic Science Research & Education (CFSRE) as a substance to monitor due to its uncontrolled status and wide availability [14].

Pentobarbital, a veterinary barbiturate used primarily for animal euthanasia and classified as a Class B Schedule 3 drug, has been found in counterfeit tablets in the US. From 2020 to 2023, it was present in 1% of 1219 seized samples [15,16]. Despite its low detection rate, pentobarbital’s high toxicity—where one gram is considered lethal—necessitates ongoing monitoring [15,16].

Carfentanil, another potent substance, was linked to 31 deaths in the UK between February and June 2017 [19], prompting the World Health Organization to recommend its inclusion under the strictest level of international control in 2017 [33].

A comparison of the manual and AI-driven thematic analyses reveals several recurring themes in social media discussions on the misuse of veterinary medications. Both approaches identified key themes, including the negative health effects of these drugs, personal experiences, polysubstance use, routes of administration, dosing, associated toxicities, and the motivations behind their misuse.

Polysubstance misuse was represented by 50 posts and comments, demonstrating the risky behaviours of people who misuse drugs. This practice is particularly dangerous due to the heightened toxicity from drug interactions, additive and synergistic effects, and increased risk of severe health implications, such as respiratory depression. The posts and comments demonstrated that veterinary drugs are often used with other drugs of misuse, with one post displaying the misuse of pentobarbital with codeine, stating that *“it was perfect to mix Nembutal and Codeine together”.* One user posted about a recent drug test result, where *“fetty tranq Xanax and mdma”* was identified.

Other posts that were analysed presented that users often took to Reddit to ask about specific combinations of veterinary drugs with other drugs of misuse, for example, *“What would happen if xylazine and K2 Spice was mixed, put into a vape pen and smoked?”* and *“what is the shrooms and carfentanil combo like?”.* In addition to this, polysubstance misuse with veterinary drugs and other veterinary drugs was apparent. This type of polysubstance misuse was mainly due to drug adulteration, where posts demonstrated xylazine, fentanyl, and medetomidine, as well as the “*Super Mario*” mixture, containing xylazine, fentanyl, diphenhydramine, heroin, and carfentanil [34].

The prevalence of posts and comments seeking advice and support on veterinary drug misuse suggests a gap in available medical guidance, prompting users to turn to online communities for information. The high number of posts related to “education/awareness” (45 posts) and “method of acquisition” (40 posts) indicates that social media serves as both a platform for raising awareness about emerging drug-related risks and a space for discussing new public health concerns. However, a key drawback is that these platforms also enable users to anonymously seek information on obtaining these substances, potentially facilitating misuse.

### 4.1. Xylazine

The analysis of social media posts and comments on xylazine misuse revealed key themes through both manual and AI-driven thematic analysis. The manual analysis identified “motivations for misuse” as the most prevalent theme, with 59 mentions. Users frequently cited xylazine’s affordability and accessibility, describing it as “*extremely cheap and easy to source*” and “*way cheaper to produce*”. Many posts highlighted its role in fentanyl combinations (“*tranq*”), with users noting that xylazine prolongs fentanyl’s effects—referred to as giving the “*fent legs*” or making the high last longer. This combination was perceived as cost effective and profitable for dealers, as it made the drug appear stronger and encouraged repeat purchases. Despite some positive posts regarding xylazine, such as *“yes, I enjoy tranq”* and *“I prefer tranq*”, the majority of experiences shared were negative, with xylazine being labelled as *“evil”* and *“poison”.*

The AI-driven analysis reinforced these findings, identifying negative health effects as the most common theme and mirroring concerns raised in the manual analysis. Discussions frequently centred on severe withdrawal symptoms and graphic descriptions of xylazine-related wounds, including extensive tissue damage, destruction of nasal and throat tissue, amputations, and severe infections [35].

Withdrawal of xylazine was a frequently discussed topic, with users often sharing their negative experiences. It was described as *“the worst withdrawal of your life”, “the worst thing I’ve ever been through”,* and *“near lethal”.* The lack of effective treatments for xylazine withdrawal or long-term therapy for xylazine addiction [36] is particularly concerning, given the severe withdrawal symptoms reported by users. The AI analysis also identified polysubstance misuse as the second most frequently discussed theme, consistent with manual observations that xylazine was used alongside fentanyl, tramadol, carfentanil, heroin, alprazolam, cocaine, and methamphetamine. This aligns with reports of xylazine rarely being identified alone, where 100% of toxicology cases in the UK detected xylazine with other common drugs of misuse [30]. Although xylazine is rarely sought out intentionally [37], posts and comments reveal that some users actively seek to obtain it, with examples such as “*where and how to obtain xylazine? Looking for info on how to obtain tranq*” and *“I am overseas looking for xylazine”.* Worryingly, users turned to social media for advice on how to use xylazine, indicating it remains a drug of choice. One user, for example, mentioned having *“just come into possession of a bottle of xylazine”* and sought guidance on *“how much to load into a syringe for a nice, regular experience.”* Moreover, the AI analysis noted public health concerns as a significant theme, aligning with user posts describing xylazine *“a huge public health issue with all the physically damaging effects of the drug”*. User testimonials on Reddit reveal both the allure and the dangers of xylazine, showing a stark contrast between its perceived benefits and the reality of its harmful impact. This discrepancy underscores the need for increased awareness and intervention to address the misuse of xylazine. The patterns observed in the discussions and experiences shared online emphasise the urgent need for targeted public health strategies and regulatory measures to mitigate the harmful effects of xylazine.

### 4.2. Carfentanil

The manual and AI-driven analyses of carfentanil-related posts yielded slightly different results. The manual analysis identified polysubstance misuse as the most prevalent theme (20 posts/comments), followed by discussions on routes and dosages (14 posts/comments) and advice and support (11 posts/comments). In contrast, the AI analysis found that carfentanil misuse was the most common theme, with potency and personal experiences ranking next. Both analyses highlighted polysubstance misuse, though in the AI analysis, it was the fourth most frequently identified theme.

Users reported instances where their drug samples, including Xanax and fentanyl, were adulterated with carfentanil. Additionally, organisations warned Reddit users about “*Super Mario*”—stamped bags containing xylazine, fentanyl, diphenhydramine, heroin, and carfentanil—which has been documented in US news reports as a public health concern [34,38]. This aligns with user posts describing their use of fentanyl, xylazine, and trace amounts of heroin and carfentanil. Carfentanil has also been found as an adulterant in counterfeit prescription opioids since 2019 [39], including falsified versions of OxyContin (oxycodone) and Xanax (alprazolam) [2,40]. Although carfentanil reports spiked in 2017 [41], a slight decline has since been observed, with 273 reported seizures in 2022, including three syringes containing both carfentanil and xylazine [2]. This is a decrease from 333 carfentanil seizures reported to the EU Early Warning System in 2021 [42].

Reddit users voiced concerns about the recent resurgence of carfentanil, with remarks like “(…) which I’m surprised to be popping back up after 5 years”, “(…) surprised it’s making a comeback”, and “(…) but there have actually been a lot of reports coming up for carfent again, surprisingly”. These posts highlight the growing apprehension expressed by users, suggesting a collective awareness of the dangers associated with carfentanil. The fact that carfentanil may be resurfacing after a period of decline indicates a troubling trend in the drug’s availability, which could lead to accidental overdoses and severe adverse effects. Carfentanil is known for its extreme potency, with it being the most potent of the commercially available fentanyl analogues [40], with these attributes being desirable to a user—“I was looking for carfentanil because it is very potent, and small doses can be used”. It was observed that users took to social media to offer advice, with comments such as “carfentanil can easily be used in humans. Obviously don’t dose it at elephant levels” and “I was addicted to IV carfentanil, so I would totally use it, however I recommend you have a tolerance first”. Others asked for guidance on Reddit, “But does carfentanil feel good?”. These examples highlight both the advantages and drawbacks of drug-related discussions on social media. The benefits of these discussions allow for the dissemination of information about dangerous adulterations, warning users to exercise caution, and potentially preventing adverse events from these new mixtures. However, it is evident that these discussions can also encourage or promote the use of these dangerous substances.

### 4.3. Medetomidine

Medetomidine was less frequently discussed in the social media analysis, with only 26 posts/comments identified through the manual analysis. This limited presence may be due to its relatively recent emergence in the illicit drug supply, with cases first reported in 2022 [12]. The manual analysis highlighted “education and awareness” as the most prevalent theme, aligning with the AI analysis, where public health concerns ranked as the second most common theme. Similar to discussions on xylazine and carfentanil, users increasingly turned to social media to voice concerns and issue warnings about medetomidine’s emergence. One post stated that *“(…) experts say the chemical, mixed into counterfeit pills and powders sold on the street, slows the heart rate to dangerous levels. It’s impossible for users to detect”*, with this post serving as a warning to others about the dangers associated with medetomidine. The misuse of medetomidine was a theme detected by the AI analysis, where one user posted about xylazine and medetomidine, stating he has *“used both substances and even though they are both alpha 2 drugs the side effects greatly vary….medetomidine can cause some side effects that are a lot worse then xylazine”*. Another user also posted about their experience with medetomidine, stating they have *“been slowly increasing my doses and I’m up to a little over 100 mcg”,* suggesting they use *“it to get the sleep, but sometimes, especially with an opiate, it’s quite nice to stay awake and read or watch tv until I pass out”*. This post illustrates the concerning trend of using this potent veterinary tranquiliser, raising safety concerns due to the casual approach to mixing it with opioids. Polysubstance misuse was identified by the dual analysis of medetomidine, where it was posted that a *“sample came back from the lab as xylazine/fent/medetomidine”*. Another post warned about *“pink fentanyl”*, which was described to *“be a veterinary anaesthetic sedative combo known as xylazine medetomidine. With severe side effects”*. This trend of combining medetomidine with other substances aligns with reports from 2022–2023, where five patients with suspected opioid overdose were found to have also used a mix of opioids (fentanyl, mitragynine, heroin, tramadol, N-pyrrolidino etonitazene), benzodiazepines (bromazolam, clonazolam, etizolam), stimulants (methamphetamine, cocaine), and others (xylazine, olanzapine, quinine, lidocaine) [43]. These findings demonstrate the complexity of substance misuse patterns, as 100% of medetomidine samples were found in conjunction with other drugs of misuse. The presence of medetomidine with multiple other illicit compounds may complicate the clinical management of overdoses and the associated withdrawal symptoms experienced afterwards.

Medetomidine must be monitored due to its emergence in drug samples in the US and Canada, where early monitoring can help to detect trends before they become widespread, allowing for timely public health responses.

### 4.4. Pentobarbital

Two of the most prevalent themes for pentobarbital were “motivations for misuse” and “methods of acquisition”, which were picked up through both analyses. Users often turned to social media to inquire about ways to obtain this drug, commonly used as a euthanasia agent in veterinary practice and previously utilised in human medicine for managing seizures and insomnia [16]. Due to its use as a euthanasia agent, a common theme encountered was people seeking to obtain it for suicidal intentions, where users stated that *“lots of people find ways to buy pentobarbital online and they use that as a “softer” means to end their life”* and that *“some people would be willing to pay thousands of dollars for a bottle of pentobarbital. It’s the gold standard for a quick and painless suicide”*. It was observed that there were links for users to buy pentobarbital online, with the post stating they *“sell pentobarbital at the best rates you will find online”.* One user posted they *“would consider the darknet”*, as they wanted to *“order 12 mL of pentobarbital”*. Similarly, it was reported by one user that *“a study in 2020 found that 47% of the vet suicides they looked at in the US used poisonings as the method and more than half of them were attributed to pentobarbital”*.

Self-poisoning with pentobarbital is not a new phenomenon, with reports documenting its online purchase [44,45]. While suicide attempts involving pentobarbital are rare [46], they may occur among individuals with access to veterinary medications. Notably, veterinary professionals face a higher suicide risk than the general population [47], with pentobarbital being the primary method used in suicide cases among this group. Beyond its intentional use in suicides, pentobarbital has recently emerged as an adulterant in drug seizures across the US, detected in 1% of samples [16]. Its presence in counterfeit tablets raises serious concerns, as users may unknowingly ingest it alongside other dangerous substances, including fentanyl, methamphetamine, xylazine, para-fluorofentanyl, and metamizole [15]. Accidental consumption poses significant health risks, such as respiratory and central nervous system (CNS) depression, particularly when combined with opioids or stimulants. The interaction of pentobarbital with opioids and benzodiazepines can amplify sedation, leading to synergistic respiratory depression [48], increasing the risk of overdose and death.

Other users took to Reddit to share their positive experiences using pentobarbital, which was described as *“glorious”* and *“it felt like the best benzo body high one could get, very warm and super anxiolytic-confidence boosting effects”*. Other positive posts and comments related to the misuse of pentobarbital included *“overall yes a very good drug lucky I got to try it”, “took 2 just slightly relaxed and no anxiety not much effect”,* and *“if I had access to more I would definitely have used them every day in high quantities*”. Alongside pentobarbital’s general misuse demonstrated through Reddit posts and comments, users also describe the polysubstance misuse they demonstrated, with comments suggesting *“pentobarbital is one of the least cyp inducing barbiturates…that’s why it was perfect to mix Nembutal and Codeine together. With both you get more relief than using just one of the drugs”*. Another user stated that they needed a *“powerful sedative”* and wrote that they *“could use fentanyl or pentobarbital + tramadol mix…this ingredients aren’t hard to get, tramadol (where I live) is a over the counter drug, pentobarbital it’s sold in veterinary shops”.*

Despite pentobarbital’s relatively low detection in recent counterfeit drug samples, its potential for toxic effects underscores the need for vigilance, where toxic doses of pentobarbital occur at approximately 1 g [48]. Healthcare professionals need to be aware of the combinational effects of pentobarbital alongside other drugs of misuse, such as opioids and xylazine.

### 4.5. Phenylbutazone and Acepromazine

The decision to analyse phenylbutazone and acepromazine stemmed from their recent detection in seized drug material [32], highlighting their potential role in illicit drug mixtures. However, the Reddit analysis revealed only six posts/comments for each substance, indicating limited discussion and awareness of these veterinary drugs on social media. For phenylbutazone, the manual analysis identified “advice/support” as the most common theme (three posts), while “motivations for misuse”, “personal experiences”, and “seizures” were each represented by a single post. In contrast, the AI-driven analysis highlighted “toxicity and adulteration” and “misuse of phenylbutazone” as the most prevalent themes. Similarly, for acepromazine, the manual analysis found “advice/support” to be the most common theme (three posts), followed by “personal experiences” (two posts) and “polysubstance misuse” (one post). The AI analysis, however, identified “misuse of veterinary drugs” as the primary theme, followed by “polysubstance misuse”. Asking for advice regarding the misuse of phenylbutazone was apparent, with one user asking what would happen *“if a human were to consume it”* and asked, *“would it also relieve pain, is it harmful, what would the negative effects be, how much should be consumed?”* Another user asked, *“what is better for recreational purposes”* and listed *“phenylbutazone (75 mg)”* in their list of drugs. Subsequently, another user presented misusing phenylbutazone for joint pains, stating, *“my joints are in a lot of pain…I’m not sure how I’m alive because I’ve been overdosing myself with Tylenol and Advil (Please don’t think I’m crazy but I’ve also taken phenylbutazone which is not available to humans it’s a horse med)”.* Phenylbutazone was once used in human medicine in the early 1950s to treat arthritis and other inflammatory musculoskeletal disorders [49], although the mid-1980s banned it due to safety concerns. Between 2016 and 2021, phenylbutazone was detected in 116 seized drug samples, frequently observed with heroin, fentanyl, and xylazine [14]. The CFSRE issued a “toxic adulterant alert” regarding its presence, raising concerns about its spread across the US, originating from Pennsylvania, similar to xylazine. It has been made clear that phenylbutazone may be present in illicit drug samples, especially those containing heroin and fentanyl, and that the adverse effects of phenylbutazone include liver and kidney damage, gastrointestinal bleeding, and blood disorders [14].

Discussions surrounding the misuse of acepromazine were evident in users seeking advice on its consumption. One user inquired, “*Can I take acepromazine? Just want to know how much/if I can take these!*” Similarly, another asked, *“Has anyone here ever taken their dog’s or cat’s acepromazine?”* These posts highlight the concerning trend of individuals turning to social media for guidance on misusing veterinary medications, underscoring the need for better awareness of the potential dangers associated with consuming drugs intended for animals. Similar to pentobarbital, one user demonstrated taking acepromazine for suicidal ideation, asking, *“if I took 4.8 g propranolol, 60 mg acepromazine, 8 g trazodone and weed as an antiemetic would I die? And how painful would it be? How long would it take?”*. This polysubstance misuse illustrates the potential for harmful consequences when veterinary drugs are misused and combined with other common drugs. Reports of acepromazine misuse are rare, though one case documented toxicity following the intentional ingestion of a pet’s acepromazine, leading to CNS and respiratory depression [50]. Due to its lack of regulation, concerns have emerged about increased availability and misuse [14], prompting some to label acepromazine as “the next xylazine”. Despite the limited data on phenylbutazone and acepromazine, enhanced monitoring and awareness efforts remain crucial. Given their potential for severe health consequences, proactive measures by health authorities, researchers, and policymakers are essential for mitigating emerging risks.

The findings of this study demonstrate that these specific drugs, aimed at animals, possess potent pharmacological properties, resulting in dangerous outcomes when taken by humans. Two of the most common themes presented within the thematic analysis were regarding the negative health effects and polysubstance misuse, demonstrating both the dangerous outcomes associated with these products, whether that is intentional or unintentional intake. The deliberate use of these animal medicines is a cause for concern, as users are actively searching for higher-potency drugs to satisfy their desires. As individuals develop dependence and tolerance to commonly misused drugs, there may be an increasing desire for more potent substances. This heightened demand for stronger drugs, including certain veterinary medications, could lead to a rise in their production and distribution to meet the evolving needs of users. Polysubstance misuse increases the exposure to multiple substances, increasing the chances of unintentional exposure to veterinary medicines. Due to the potency of animal medicines, such intentional consumption can lead to severe and unpredictable health outcomes. Results from the study demonstrate that adulteration using veterinary medicines is present within a wide range of illicit drugs, such as alprazolam, methamphetamine, heroin, and fentanyl. As adulteration using veterinary medicines increases, the potential harm beyond a single demographic of drug users broadens.

The findings of this study have several practical implications for public health policies and veterinary drug regulations. Policymakers should enhance surveillance systems, particularly using AI tools, to quickly detect emerging trends in veterinary drug misuse across platforms like Reddit. Regulatory frameworks should be reassessed to address the increasing presence of veterinary drugs in illicit markets. Public awareness campaigns should target at-risk populations, and cross-agency collaboration is crucial to managing misuse. AI significantly strengthens the robustness of the findings by efficiently processing large datasets and identifying patterns missed in manual analysis, such as the combination of veterinary drugs with traditional illicit substances and shifts in language surrounding misuse. Despite its limitations, AI’s predictive capabilities further support proactive public health strategies, enabling timely interventions.

## 5. Limitations

While this study offers valuable insights into online communities and behaviours related to veterinary drug misuse, several limitations should be acknowledged. First, social media users do not fully represent the broader population of drug users, as many individuals do not share their experiences online, leading to an incomplete understanding of offline behaviours. Additionally, the pseudonymous nature of Reddit participation makes it challenging to accurately assess demographic information, as users are not required to disclose personal details such as age or gender, limiting the ability to contextualise findings across different demographic groups [21].

Furthermore, during preliminary research, no street names for pentobarbital, medetomidine, phenylbutazone, or acepromazine were identified. However, it was later discovered that “Nembuterol” was a slang term used for pentobarbital in online discussions. This oversight may have resulted in the exclusion of data related to pentobarbital misuse.

This study also relied on data from anonymous social media accounts, which can sometimes contain inaccurate or deliberately misleading information. While AI analysis filtered out posts with terms related to suicide, manual analysis did not apply this filter, potentially including misleading content. Moreover, the AI tool used for analysis was not specifically trained on this dataset or subject matter, which may have led to categorising posts based on the AI’s own criteria, potentially introducing biases or misattributions. However, the manual analysis enabled to the identification of AI-induced bias.

Additionally, as an exploratory analysis without statistical testing or hypothesis testing, the findings should be considered preliminary. Despite these limitations, the study highlights emerging trends in veterinary drug misuse and provides a foundation for informing relevant policies and interventions aimed at reducing drug-related harm. Future research could address these limitations by expanding the scope to multiple social media platforms and incorporating statistical analyses for more robust conclusions. This work serves as a stepping stone for more comprehensive, confirmatory research.

## 6. Conclusions

This study has highlighted emerging trends in veterinary drug misuse, as discussed on Reddit, focusing on six veterinary drugs identified in previous research [28,29]. Through the analysis of posts and comments, this study examined factors such as adverse effects, routes of administration, doses, polysubstance misuse, acquisition methods, and motivations for misuse. By combining manual and AI-driven thematic analysis, this research provides a comprehensive and accurate understanding of social media discussions around veterinary drug misuse. These findings show the potential of social media as an early-warning tool for identifying emerging drug misuse trends, offering valuable insights that can inform public health policies and intervention strategies. Moving forward, future research should incorporate a wider range of data sources, including other social media platforms, to capture a broader demographic and avoid limitations to one site. Additionally, policymakers should consider integrating social media monitoring into public health surveillance systems to enable real-time detection and response to evolving drug misuse patterns. While this study reflects only the experiences shared by social media users and may not represent the wider population, it is essential to continue monitoring substances like xylazine, medetomidine, carfentanil, and pentobarbital, which are increasingly misused, and to remain vigilant regarding phenylbutazone and acepromazine, which, despite not being commonly misused, have recently appeared in the illicit drug supply.

## Figures and Tables

**Table 1:** List of drugs and their corresponding keywords used in the Apify tool for automated extraction of relevant posts and comments. Keywords were carefully selected to ensure comprehensive coverage of content related to the misuse of these substances.

Drug	Keywords Used in Apify
Xylazine	“xylazine”, “tranq”, “tranq dope”, “zombie drug”
Carfentanil	“carfentanil”, “carfentanyl”, “carf”, “wildnil”, “serial killer drug”, “drop dead drug”
Medetomidine	“medetomidine”
Phenylbutazone	“phenylbutazone”
Pentobarbital	“pentobarbital”
Acepromazine	“acepromazine”

**Table 2:** Inclusion and exclusion criteria for Reddit posts, detailing the protocol used to determine which posts were considered or excluded from the analysis.

Inclusion Criteria	Exclusion Criteria
Posts and comments posted in English	Posts and comments posted in a language other than English
Posts and comments discussing the use/misuse of the specified veterinary products	Content focused solely on veterinary use without any reference to human consumption
Publicly available posts and comments	Private or restricted-access posts and comments
Posts and comments including information regarding the human consumption of the specific drugs	Posts discussing unrelated substances or general drug use without mentioning the specified veterinary drugs
Posts and comments from any region, regardless of the user’s age, gender, religion, or race (if this information was provided in the post or comment)	Duplicate posts or comments already included in the dataset
Posts and comments describing/discussing the dose/route/method of acquisition of the specified drugs	Posts and comments that mention the drugs of interest without providing relevant context or substantive information

**Table 3:** Summary of identified themes and corresponding post examples for drug-related discussions.

Drug Name	Themes and Example Reddit Posts
	**1. Motivation for Misuse**
Xylazine	*e.g., (…) so you get that shouldn’t be injected in humans constantly, like a drug addict does, that is extrememly cheap and easy to source*
	*e.g., Fent and xylazine are wayyyy cheaper to produce and have flooded the US from China.*
	*e.g., And people are doing it purposefully. “Tranq gives fent legs”, it helps fent subjectively “last longer” as heroin users switch to fent, which has a shorter half life.*
	*e.g., Fent high is significantly shorter than heroin. The xylazine cut extends that high so you don’t have to keep hustling non-stop. Ultimately, it is more profitable because it is more popular.*
	*e.g., (…) You get the brief window of being high from the fent, then you stay zoned out for hours later like a zombie from the Xylazine. No real high, you’re just incredibly tranquilized, but you feel more like you got your money’s worth.*
	*e.g., Yes I enjoy tranq.*
	*e.g., It’s get too a point where even pure fentanyl doesn’t do nothing too you so I just look at tranq as the next step if you wanna still get high so yea I enjoy it now I just tell my dealer don’t put too much or I buy it separately and do small portions I’m in the tranq capital btw.*
	*e.g., Dealers are lacing the fentanyl with xylazine nowadays. It makes the user nod harder, so they end up thinking the dope is really strong and will buy from them again*
	*e.g., (…) dealers who are now selling tranq as a stand alone drug because so many people are addicted to it that they are requesting it*
	*e.g., I’ve always wanted to try xylazine on its own*
	*e.g., (…) And tbh….I prefer xylazine….whe asking what stamp is hot on the bloxk…i ask for heavy trank. The shots I did which I believe were mostly trank (xylazine) it was like ketamine….*
Carfentanil	*e.g., (…) I was looking for Carfentanil because it is very potent, and small doses can be used*
	*e.g., (…) apart from the fact that I am violently addicted to injecting heron cut with carfent but it’s basically a plant so it doesn’t really affect me in any bad way*
	*e.g., (…) Carfentanyl alone is shit just like regular fentanyl. It just knocks you out with no euphoria. That’s why I mix it with regular H. So I can feel it.*
	*e.g., Carfentanyl is the most euphoric substance I have ever had. You haven’t had carfentanyl in your possession if you claim it knocks you out without euphoria. I had highly pure carfentanyl oxolate. Carfent alone is good shit…*
	*e.g., I need some of that carfentanyl right now.*
Medetomidine	*e.g., Dope is now being found with medetomidine which is similar to xylazine. I guess now that states are banning xylazine they have now switched to this crap.*
Pentobarbital	*e.g., (…) a study in 2020 found that 47% of the vet suicides they looked at in the US used poisoning as the method and more than half of them were attributed to pentobarbital*
	*e.g., (…) I’ve been looking into pentobarbital as a method of finally putting myself out of my misery, but it’s damn near impossible to find*
	*e.g., (…) Pentobarbital is considered to be more pleasent and abuseable.*
	*e.g., Some people would be willing to pay thousands of dollars for a bottle of pentobarbital. It’s the gold standard for a quick and painless suicide.*
Phenylbutazone	*e.g., (…) My joints are in a lot of pain and I have to wait over 3 months for an appointment with a doctor. In the meantime I’m not sure how I’m alive because I’ve been overdosing myself with Tylenol and Advil. (Please don’t think I’m crazy but I’ve also taken phenylbutazone which is not available to humans it’s a horse med)*
	**2. Personal Experiences/Public Perception**
Xylazine	*e.g., (…) Xylazine is, and I rarely use this word, evil.*
	*e.g., That’s all there is out in my area anymore fentanyl cut with tranq …I hate tranq*
	*e.g., (…) And you’ll KNOW when you get regular fetty once you’re addicted to tranq because you will go into the WORST withdrawal of your LIFE in no time. xylazine sickness cold turkey is the worst thing I have ever been thru in my 29 years*
	*e.g., (…) xylazine should NEVER be in the human body, and the withdrawals it causes last weeks and are the worst thing I’ve ever been through in my life*
	*e.g., The withdrawal for Trang aka xylazine is horrendous I speak from experience. It is the worst thing I ever went through*
Carfentanil	*e.g., I thought carfent was stronger than fent ive overdosed on both*
	*e.g., (…) I’ve come to the conclusion that a lot of the strong shit I’ve been getting lately has been partly or entirely carfentanil. Tastes basically the same as regular fetty but is way stronger. I’ve asked dealers their opinions and they’ve agreed*
Medetomidine	*e.g., (…) Medetomidine can cause some side effects that are alot worse then xylazine. They are still both poison though.*
	*e.g., Medetomidine is just a anesthestic/analgesic it’s not that dangerous unless you’re a drug addict taking random cocktails*
	*e.g., (…) I had a sample tested but ironicslly it was like THE BEST dope I’ve ever seen it had that met stuff in*
Pentobarbital	*e.g., Only experience ive had is with pentobarbital, and yea, it sucked.*
	*e.g., (…) Pentobarbital is a slightly more intense high overall and lasted for a longer duration of time too*
	*e.g., As someone who has used both, phenobarbital is SHIT and pentobarbital is the holy grail*
	*e.g., (…) i want to try pentobarbital once in my lifetime*
	*e.g., (…) pure Pentobarbital is absolutely blissful, I had the chance to try it one year ago*
	*e.g., Pentobarbital is beyond awesome. Have fun*
	*e.g., I happen to love the way Pentobarbital (Nembutal) makes me feel. I take prescribed Xanax multiple times a day. Nembutal hits hard and fast you will feel and seem basically drunk but with a tingly body and complete relaxation*
	*e.g., Honestly I loved pentobarbital way to much I would take it and just flat out cold pass out for 4 h straight*
	*e.g., I tried new veterianry pentobarb and it was GLORIOUS…it felt like the best benzo body high one could get, very warm and super anxiolytic-confidence boosting effects. As for the negative side, it was just too addictive, as soon as I got it I began using every day…*
	**3. Adverse Effects**
Xylazine	*e.g., (…) I’m still dealing with blisters and tissue damage in my nose and throat. Pretty sure it fucked my molars up too. Stuff is a poison and will destroy any part of your body you put it into*
	*e.g., (…) And traq wds can kill now with the dehydration and insane blood pressure and irregular heart beats this shit causes.*
	*e.g., (…) that’s another thing the tranq makes me have vivid lucid dreams even nightmares like really weird sleep paralysis too sometimes*
	*e.g., My mom nearly lost her whole entire leg due to xylazine. She has had several grafts. It has taken nearly two years to get to a stable healing point. It had also started eating holes in her heart. She is very lucky to have survived through all of it and it’s because she got and stayed sober. If it doesn’t cause an OD it WILL eventually cause severe health issues.*
	*e.g., (…) the tranq is making their bodies shut down; slow breathing, lethargy, low heart rate, but they’re resisting the natural urge to sleep in that state because going to sleep wastes the high. So they stand there, barely awake, while their whole bodies shuts down, fighting the urge to nod out*
	*e.g., (…) Could never breathe out of my nose, and it was always hitting, and bleeding. Even after I stopped, it took months for my nose to heal, and it’s still not as open as it was before.*
Carfentanil	*e.g., Carfentanil had me literally hallucinating/tripping. Almost like fever dreams where you’re nodded and just seeing the craziest shit.*
	*e.g., I was doing these bags before it hit the news. They literally knocked me out.*
	**4. Route/Dose/Appearance**
Xylazine	*e.g., (…) I smoked my tranq and i ended up having a bunch of black sludge come out of my lungs.*
	*e.g., Well I’m trying to snort it I’ve snorted it twice today but people are scaring me cuz it can fuck their nose*
	*e.g., Can xylazine cause those sores if you don’t shoot, smoke or sniff? The only consumption method I use is orally.*
	*e.g., (…) I was only snorting it, and it still decided to eat my legs up!*
	*e.g., Xylazine is only “flesh eating” if injected. It can be reltively safely taken orally to taper but it’s almost impossible to determine what dose you’d need without knowing how much was in your dope.*
Carfentanil	*e.g., A few micrograms of carfentanil will help.*
	*e.g., Was surprised to learn that carfentanil is the safest opiate there is, since the multiple of a therapeutic dose needed for an overdose is so high. So, if you can accurately measure it, you’d need to ’accidentally’ put like 5 times the amount you planned into someone to cause OD.*
	*e.g., I smoke about 2 g of street fentanyl daily but I hate the way carfentanyl taste but I would start at about a tenth of gram daily*
Medetomidine	*e.g., (…) I’ve been slowly increasing my doses and I’m up to a little over 100 mcg*
Pentobarbital	*e.g., Do it orally if you do 200 mg is a very nice dose*
	*e.g., (…) 75–150 mg Pentobarbital is quite nice orally with onset at 35 min, peak hits at 40 -45 min.*
	*e.g., I feel that oral route would be risky as first pass metabolism is really unpredictable. It would feel like a slow death. IV pentobarbital would be quick and easy.*
	*e.g., If I had had access to more I would definitely have used them every day in high quantities*
	**5. Polysubstance Use**
Xylazine	*e.g., (…) Lab analysis showed: Lidocaine, Fentanyl, Tramadol, DXM, Niacinamide, Xylazine Experience Note: ‘Knocked out in one minute, \ [non-fatal\ ] overdose’*
	*e.g., Yea, my last near fatal OD the dope did not only have xylazine in it, it also had carfentanil in it and two other fentanyl analogues that were all several times more potent than just actual fentanyl. As If just straight fentanyl wasn’t already way too potent itself. It did however contain some amount of diacetylmorphine (actual heroin) in it also. Cocaine too which boggles my mind*
	*e.g., (…) Also when I toom drug test I came up for fetty tranq Xanax and mdma. All I was doing was the fetty powder. Stuffs horrible*
	*e.g., (…) When you mix xlazine with opiates like heroin or fentanyl they work together (synergize) and make the user feel much, much higher. The mixture makes people act and look just like zombies.*
	*e.g., What would happen if xylazine and K2 Spice was mixed, put into a vape pen and smoked?*
	*e.g., Has anyone done tranq-dope with mdma? Any side effects? I want to take molly for new years but I do tranq-dope and idk if that’s gonna cause bad side effects. Has anyone done it and how was your experience?*
Carfentanil	*e.g., (…) It doesn’t do anything but that’s because I was doing fentanyl, xylazine and “trace amounts” of heroin and carfentanil.*
	*e.g., i overdosed on laced xanax and my pee tested positive for carfentanil.*
	*e.g., (…) SMURF DOPE:“It has vibrant blue color. Either methamphetamine or heroin that has been laced with fentanyl. Perhaps not just fentanyl but carfentanil”, said Brad Brewer, a Harm Reduction Specialist with the Kentucky River District Health Department.*
	*e.g., My very last near fatal OD, and the last time I used, was in mid 2019. I bought it as “heroin” supposedly but of course I knew better. Once what I had left of that bag was tested it came out to be, in this order, furanylfentanyl, Acrylfentanyl, Carfentanil, Remifentanil, diacetylmorphine (acetylated morphine) 4-ANPP, cocaine, xylazine, and mannitol.*
	*e.g., Carfentanyl + clonazolam should do the trick.*
	*e.g., Drug users of Reddit, what is the shrooms and carfentanil combo like?*
	*e.g., NY Public Health Alert: “Super Mario” stamped bags contain xylazine, fentanyl, DPH, heroin, carfentanil*
Medetomidine	*e.g., Sample came back from lab as xylazine/fent/medetomidine*
	*e.g., mixing a dangerous chemical sedative called medetomidine into fentanyl*
	*e.g., (…) they found she had butorphanol, azaperone, and medetomidine in her system.*
	*e.g., Dope is now being found with medetomidine which is similar to xylazine. I guess now that states are banning xylazine they have now switched to this crap*
Pentobarbital	*e.g., (…) Pentobarbital is one of the least cyp inducing barbiturates as apposed to Seconal for instance. That’s why it was perfect to mix Nembutal and Codeine together.*
	*e.g., I need: a powerful sedative, a neuromuscular blocker, a potassium ion-donating agent. For the sedative I could use fentanyl or pentobarbital + tramadol mix.*
Acepromazine	*e.g., What I want to know is if I took 4.8 g propranolol, 60 mg acepromazine, 8 g trazodone and weed as an antiemetic would I die? And how painful would it be?*
	**6. Advice/Support**
Xylazine	*e.g., I’m developing gross, necrotic-looking, very, very itchy xylazine ulcers all over my legs. More so, in injection sites where a “miss” took place. My skin itches like crazy, flakes, and these lesions form. Anyone else experience this phenomenon?*
	*e.g., Yes I know this is a fent group but idk where else to ask… I only hear bad things about tranq… No euphoria, skin lesions, blacking out, horrible withdrawals… does anyone actually like tranq dope and actively search it out???*
	*e.g., I swear everything in my area and surrounding areas is flooded and I mean FLOODED with nasty tranq dope! And I mean I hate it like to the point where I really don’t wanna get high anymore. It’s such a unenjoyable high. Anyone else feel like this ?*
	*e.g., I just came into possession of a bottle of xylazine and I was wondering how mich I load into a syringe for a nice, regular experience?*
Carfentanil	*e.g., I was addicted to IV carfentanil, so I would totally use it, however I recommend you have a tolerance first!*
	*e.g., (…) so if ur gonna reduce the ham, you need to provide alternatives, like carfentanyl, which has been proven to not be addictive and also cure cancer and hiv. so stop with the weed, and reduce ham*
	*e.g., I wonder if carfentanil (trace) would be deadly. I have no idea how much “trace” is.*
Pentobarbital	*e.g., any information on these and what to expect? (pentobarbital)*
	*e.g., You’re right, I think pentobarbital would do the trick!*
Phenylbutazone	*e.g., Toxic dose phenylbutazone What would a toxic dose of bute be? Also what would be symptoms and treatment in a human? Just curious*
	*e.g., I was wondering what does a horse drug such as phenylbutazone aka “bute” or also called equipalazone do if a human were to consume it? Would it also relive pain, is it harmful, what would the negative effects be? How much should be consumed?*
Acepromazine	*e.g., Has anyone here ever taken their dog’s or cat’s acepromazine?*
	*e.g., Can I take Acepromazine? So I recently ran into a couple of 25 mg pills of acepromazine…just want to know how much/if I can take these! Any advice helps.*
	**7. Awareness/Education**
Xylazine	*e.g., (…) Once using regularly the odds of fatality exponentially increase due to OD, open sores causing infections, or related complications such as malnutrition/dehydration that occur with substance abuse. There is no “safe” dose of this approved for human comsumption.*
	*e.g., (…) High doses cause individuals to become nonresponsive via a shutdown of the CNS and overdoses can be fatal. It does NOT respond to narcan. They are, however, producing xylazine test strips that I highly recommend any users purchase or get ahold of. Many community harm reduction groups are giving them out as well. Please, guys, test your supply every time.*
	*e.g., The ease of access to Fentanyl and its analogs, along with xylazine, is truly alarming…why isnt this a global issue?*
	*e.g., Xylazine is not safe*
	*e.g., Fake tranq accords going around the UK….my friend bought 100 boxes of what appeared to be accord codeine phosphates….i was having weird hallucinations and felt incredibly weird and sleepy….i sent them to a lab to get tested and got xylazine test strips…they were just xylazine and nothing else.*
Carfentanil	*e.g., I live in Cuyahoga county now, but just a heads up that carfentanil is making a comeback out our way. If you know anyone doing any type of hard drugs, please encourage them to at least get a test kit for their stuff.*
	*e.g., Also trace amount of carfent lol which I’m surprised to be popping back up after 5 yrs*
	*e.g., (…) but there have actually been a lot of reports coming up for carfent again, surprisingly.*
Medetomidine	*e.g., (…) Experts say the chemical, mixed into counterfeit pills and powders sold on the street, slows the human heart rate to dangerous levels. It’s impossible for drug users to detect*
	*e.g., (…) The one they’re warning about now is fentanyl cut with medetomidine, which is a surgical analgesic. Different, but still devastating.*
	e.g., “PINK FENTANYL” Batches are popping up all around Ohio!—A Chunky Powdery substance, likely colored in some variation of Pink & thought to be packaged in paper. Altho… Contains little to no Fentanyl. Suspected to be a *Veterinary anesthetic|sedative substance combo*. Known as *Xylazine|Medetomidine*. With **severe** side effects!
	**8. Method of Acquisition**
Xylazine	*e.g., Do you know where I can find it any serious people hit me up I am overseas looking for xylazine*
	*e.g., You can buy a lot of large animal drugs online, from catalogues, and in feed stores—including xylazine. It’s actually kinda horrifying how many prescription drugs you can just buy in feed stores without a prescription.*
	*e.g., Where and how to obtain xylazine? Looking for info on how to obtain tranq*
	*e.g., Willing to pay high in the five figures to a clandestine chemist that can produce xylazine*
Carfentanil	*e.g., Was it like really pure shit? Where do u even find carfentanil at Darkweb*
	*e.g., (…) I was looking on the darknet some days ago and I came across the listing on a market of “Carfentanil pure powder” 50 g for 1000 $, I messaged the seller and he said that he can send me 1 g for 50 $ or 3 for 90 $.*
	*e.g., Where does one get this carfentanil or fentanyl stuff?*
Pentobarbital	*e.g., Honestly, I’m SO jelous of everyone who got Pentobarbital. Despite I generally cheer for people.* *Those who managed to get it and don’t use it …* *LET ME BUY IT FROM YOU AND USE IT MYSELF.*
	*e.g., (…) I’m thinking of ordering pentobarbital, the drug they used on him, online.*
	*e.g., I just want a place where I can get pentobarbital delivered that’s all*
	*e.g., (…) These popular media reports of pentobarbital being a peaceful method of suicide have led to increased interest in obtaining it from jurisdictions where it is less regulated.*
	*e.g., Is pentobarbital available for purchase from pet stores in Tijuana? If not where should I look?*
	*e.g., (…) I’d like to know how to get my hands on some Pentobarbital.*
	*e.g., (…) Mypeacefulend.com are the leading supplier of nembutal and other barbiturates online.*
	**9. Seizures**
Carfentanil	*e.g., A dealer in drugs and guns sentenced to 20 years after the country’s largest seizure of the deadly drug carfentanil has been granted bail by a judge of Ontario’s highest court, pending an appeal. Maisum Ansari was convicted in February of possession for the purpose of trafficking after police seized 33 firearms and 26.5 kg of carfentanil from a basement apartment he owned.*
Phenylbutazone:	*e.g., (…) In a review of case data from NMS Labs from 2016–2021, 116 seized drug samples from Pennsylvania were identified as containing phenylbutazone.*

**Table 4:** A Summary table highlighting the top themes identified for each drug through AI analysis.

Drug	Top 5 Occurring Themes	Post Examples
Xylazine	Negative Health Effects/Consequences Poly Substance Use Public Health Concerns Addiction/Withdrawal/Dependence Public Perception/Experiences	*e.g., “(…) It’s primarily the Xylazine that causes these people to sleep on the sidewalk or walk about in a stupor, the Xylazine is also responsible for the horrendous skin abscesses that rapidly progress into full blown infections often leading to amputation”.* *e.g., “ (…) My mom nearly lost her whole entire leg due to xylazine. She has had several grafts. It has taken nearly two years to get to a stable healing point. It had also started eating holes in her heart”.* *e.g., “ (…) we found out later it was meth, xylazine, and fentanyl”* *e.g., “(…) it’s causing homelessness, open drug use in some parts of Philly, and a huge public health issue with all the physically damaging effects of the drug”* *e.g., “(…) These things that are * *flooding the streets scare me more than coke or heroin: this is going to be devastating.”* *e.g., “ (…) The problem is how fucking bad you feel when you aren’t, so you keep chasing just desperately trying to escape how shit you feel when you’re off the crap.”* *e.g., “(…) xylazine should NEVER be in the human body, and the withdrawals it causes last weeks and are the worst thing I’ve ever been through in my life”* *e.g., “I’m developing gross, necrotic-looking, very, very itchy xylazine ulcers all over my legs. More so, in injection sites where a “miss” took place. My skin itches like crazy, flakes, and these lesions form. Anyone else experience this phenomenon?”*
Carfentanil	Drug Misuse Potency Personal Experiences Public Health Concerns Poly Substance Use	*e.g., “ I was doing these bags before it hit the news. They literally knocked me out.”* *e.g., “I was looking for Carfentanil because it is very potent, and small doses can be used and because it had been studied pretty well”* *e.g., “ Got carfentanyl thinking it was regular fentanyl. Just told it was stronger. Woke up hours later without being narcanned somehow because I was alone. I should have been dead. No idea how I’m still here.”* *e.g., “ Didn’t know carfent was being found more frequently as of recently.”* *e.g., “(…)SMURF DOPE: “It has vibrant blue color. Either methamphetamine or heroin that has been laced with fentanyl. Perhaps not just fentanyl but carfentanil””*
Medetomidine	Misuse of Medetomidine Public Health Concerns Lack of Antidote or Detection Poly Substance Use Need for Awareness and Education	*e.g., “(…) have personally used both substances and even though they are both alpha 2 drugs the side effects greatly vary but of course they do have some similar side effects. Medetomidine can cause some side effects that are alot worse then xylazine.”* *e.g., “Dope is now being found with medetomidine which is similar to xylazine. I guess now that states are banning xylazine they have now switched to this crap”* *e.g., “(…) Experts say the chemical, mixed into counterfeit pills and powders sold on the street, slows the human heart rate to dangerous levels. It’s impossible for drug users to detect.””* *e.g., “ (…) The sedative was found in combination with opioids such as fentanyl, nitazenes and heroin, as well as with tranq and the anti-anxiety drug alprazolam (Xanax).”* *e.g., “(…) no one knows what long-term health effects this new cocktail of chemicals will cause in the human body.”*
Pentobarbital	Motivations for Misuse Personal Experiences Acquisition of Drugs Poly Substance Use Public Perception	*e.g., (…) a study in 2020 found that 47% of the vet suicides they looked at in the US used poisoning as the method and more than half of them were attributed to pentobarbital* *e.g., I want to get access to pentobarbital and sodium thiopental, to take it and die.* *e.g., (…) pure Pentobarbital is absolutely blissful, I had the chance to try it* *e.g., I tried new veterianry pentobarb and it was GLORIOUS…it felt like the best benzo body high one could get, very warm and super anxiolytic-confidence boosting effects. As for the negative side, it was just too addictive, as soon as I got it I began using every day* *e.g., Lots of people find ways to buy pentobarbital online and they use that as a “softer” means to end their life.* *e.g., I need help. I need to order 12 mL of pentobarbital. I would consider the darknet but idk how to trust what i get. Anyone know of a serious supplier? Thanks in advance.* *e.g., (…) I’m thinking of trying to go to Tijuana and getting pentobarbital and maybe Xanax and heroin too* *e.g., Pentobarbital these days is pretty popular among those with terminal illness*
Phenylbutazone	Toxicity and Adulteration Misuse of Phenylbutazone Comparative Analysis with Other Drugs Curiosity and Inquiry into Drug Effects Potential Damage and Health Implications	*e.g., (…) 116 seized drug samples from Pennsylvania were identified as containing phenylbutazone* *e.g., (…) I’ve also taken phenylbutazone which is not available to humans* *Simple google search shows phenylbutazone is not good for humans. Ketamine should not be referred to as a horse drug as it is widely and regularly used in human medicine.* *e.g., I was wondering what does a horse drug such as phenylbutazone aka “bute” or also called equipalazone do if a human were to consume it? Would it also relive pain, is it harmful, what would the negative effects be? How much should be consumed?* *e.g., (…) The serious adverse effects of phenylbutazone can include gastrointestinal bleeding, liver and kidney damage, and blood disorders*
Acepromazine	Misuse of Veterinary Drugs Polysubstance Use Mental Health and Suicidal Ideation Lack of Knowledge and Uncertainty About Safe Drug Use Negative Health Impacts	*e.g., Has anyone here ever taken their dog’s or cat’s acepromazine?* *e.g., What I want to know is if I took 4.8 g propranolol, 60 mg acepromazine, 8 g trazodone and weed as an antiemetic would I die?* *e.g., Can I take Acepromazine? So I recently ran into a couple of 25 mg pills of acepromazine…just want to know how much/if I can take these! Any advice helps.* *e.g., (…) It can cause significant organ damage in humans.*

## Data Availability

The original contributions presented in this study are included in the article. Further inquiries can be directed to the corresponding author.
